# Occult ovarian high-grade serous carcinoma presenting as isolated inguinal lymph node metastasis: A case report and literature review

**DOI:** 10.1016/j.tranon.2025.102371

**Published:** 2025-04-02

**Authors:** XiaoJing Guan, Zhi Ma, JianHua Yang

**Affiliations:** Department of Gynecology and Obstetrics, Sir Run Run Shaw Hospital, Zhejiang University School of Medicine, Hangzhou 310016, China

**Keywords:** Ovarian cancer, Superficial inguinal lymph node, Lymphatic metastasis, Mechanism

## Abstract

Superficial inguinal lymph node (SILN) metastasis is rare in ovarian cancer, particularly as an isolated presentation without peritoneal dissemination. Here we report a case of solitary SILN metastasis in a patient with high-grade serous carcinoma of ovarian cancer and review previously reported cases from literature. A 58-year-old woman presented with isolated right groin swelling (approximately 4 cm), excisional biopsy suggested ovarian origin but comprehensive imaging and laparoscopy failed to identify a primary intra-abdominal tumor, and postoperative pathology confirmed no evidence of ovarian involvement. Following multidisciplinary consultation, the patient received six cycles of paclitaxel (175 mg/m²) plus carboplatin (AUC-5) chemotherapy (21-day intervals). Remarkably, five years post-treatment, she remains disease-free, highlighting the potential for favorable outcomes even in rare metastatic presentations. This case demonstrates that ovarian cancer is not a disease confined to the intra-peritoneal cavity; SILN metastasis might occur in rare cases possibly via lymphatic and/or hematogenous spread route under specific circumstances. Further investigations related to risk factors and metastatic patterns are warranted to explore the mechanisms and clinical implications of isolated SILN metastasis in ovarian cancer.

## Highlights

• Isolated inguinal lymph node metastasis can be a rare initial sign of ovarian cancer.
• The metastasis may occur via potential inguinal lymphatic and/or hematogenous route.
• Patients with isolated inguinal metastasis live longer than other stage IV patients.
• The timing of surgery and whether neoadjuvant chemotherapy necessary is disputable.

## Graphical abstract

**Figure:**
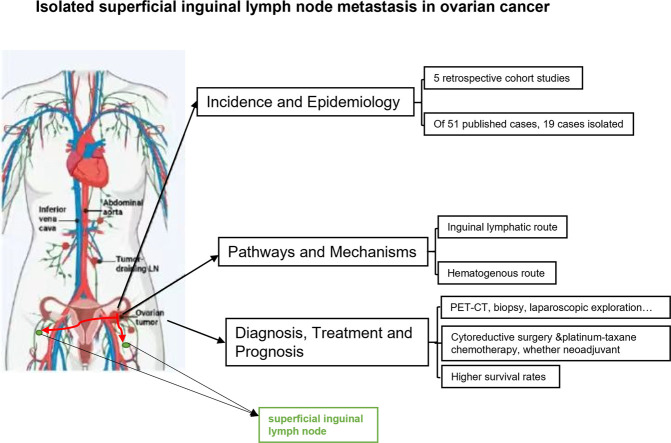

## Introduction

Ovarian cancer is a common gynecological malignancy ranking third in incidence after cervical and endometrial cancers, but represents the leading cause of death among gynecological malignancies [1]. Over 70 % of patients with ovarian cancer were diagnosed at an advanced stage, as International Federation of Gynecology and Obstetrics (FIGO) stage III or IV [2], due to its insidious onset and the lack of early detection methods. Nowadays, Carbohydrate Antigen 125 (CA125) and Human epididymis protein 4 (HE4) are the only approved serum biomarkers for use in ovarian cancer, however not sufficient for early detection. Multivariate index (MVI) assays have been developed to mitigate the limitations of single serum biomarkers in the diagnosis of ovarian cancer. The Risk of malignancy algorithm (ROMA) integrates menopausal status, CA125 and HE4 concentrations to diagnose women with a pelvic mass, showing higher sensitivity and specificity [3]. Lymphatic spread and peritoneal dissemination constitute the main metastatic routes in ovarian cancer. Three primary lymphatic metastasis have been described: (1) along infundibulopelvic ligament to paraaortic and paracava lymph nodes, (2) via subovarian plexus to the obturator and pelvic iliac lymph nodes, and (3) through the bilateral round ligament to the external iliac and deep inguinal lymph nodes, which finally drained towards the superficial inguinal lymph nodes (SILNs). SILN metastasis may occur concurrently with para-aortic or pelvic lymph node metastasis or in isolation.

While ovarian cancer with SILN involvement was historically classified as stage IIIC, the 2014 FIGO staging system reclassified it as stage IVB [2], though its prognostic value remains controversial. Cases of occult ovarian cancer presenting initially with SILN metastasis are exceedingly rare. We report such a case and review the literature regarding epidemiology, mechanisms, prognosis, and management of isolated SILN metastasis in ovarian cancer.

## Case report

In March 2019, a 58-year-old Chinese woman presented with a one-month history of painful right inguinal swelling. The subcutaneous mass gradually enlarged to about 4 cm despite initial antibiotic treatment at local hospital. Excisional biopsy revealed metastatic adenocarcinoma with necrosis and calcification, suggesting a possible origin from the ovary or other female reproductive organs. Immunohistochemistry showed: Ckpan (+++), CK7 (+++), CK20 (-), CA125 (+++), CEA (focal +), TTF-1 (-), NapsinA (-), CDX2 (-), p53 (+), Ki-67 (about 30 %). Extensive imaging, including transvaginal ultrasonography, cranial, thoracic, and abdominal CT scans, as well as gastroscopy and colonoscopy showed no primary tumor. The patient prefered to our hospital for further diagnosis and treatment. Physical examinations and serum tumor markers (CA125, CA199, and CEA) were unremarkable. Ultrasound of axillary, supraclavicular, cervical, pelvic, and retroperitoneal lymph nodes showed no significant enlargement. Transvaginal ultrasound revealed an endometrial thickness of 0.87 cm, with no abnormal masses in the adnexal regions. Positron emission tomography-computed tomography (PET-CT) showed: (a) patchy, high-density areas in the right inguinal region with increased 18-fluorodeoxyglucose(18F-FDG) uptake, considered post-operative changes; (b) several small lymph nodes in both inguinal regions without abnormal FDG uptake, possibly chronic inflammatory lymphadenitis; (c) patchy FDG uptake in the uterine cavity, recommending gynecological examination to exclude lesions; (d) no abnormal FDG metabolism in other areas. Review of the biopsy confirmed metastatic adenocarcinoma in the inguinal lymph node with extensive necrosis and calcification, consistent with metastatic ovarian serous carcinoma. Immunohistochemistry results: CK20 (-), CK7 (+), CDX-2 (-), ER (+), PR (-), PAX-8 (+).

Due to the unknown primary tumor location and pathology suggesting a female reproductive system origin, the patient underwent diagnostic curettage, laparoscopic bilateral salpingo-oophorectomy, and multiple peritoneal biopsies. Laparoscopy revealed no ascites or macroscopic evidence of abdominopelvic disease. The left ovary had a 1.5*1.5 cm cyst, while the right ovary was slightly atrophic. Both fallopian tubes appeared normal. Intraoperative pelvic lavage cytology showed a few atypical cells. Frozen sections and postoperative pathologic diagnosis of bilateral adnexectomy specimens, endometrial tissue and peritoneal tissue showed no tumor tissue. As the primary tumor site remained unclear postoperatively, a multidisciplinary consultation was held. Experts from gynecology, pathology, surgical oncology, medical oncology, and radiation oncology concluded that the diagnosis was "metastatic carcinoma to the inguinal region, primary unknown." They recommended further pathological sampling and subsequent treatment with systemic chemotherapy (taxane plus platinum) and inguinal radiotherapy. Additional pathological sampling of the bilateral ovaries and fallopian tubes, after complete embedding, still showed no tumor. The patient was subsequently treated with six cycles of paclitaxel (175 mg/m²) plus carboplatin (AUC-5) chemotherapy at 21-day intervals, with the last cycle on September 9, 2019. Follow-up evaluations, including regular imaging and tumor marker assessments have shown no signs of tumor recurrence. Five years later, the patient remains disease-free, underscoring the efficacy of systemic chemotherapy in managing isolated inguinal lymph node metastasis in the context of occult ovarian carcinoma.

## Discussion

### Incidence and epidemiology of SILN metastasis in ovarian cancer

Lymph node metastasis is one of the most common routes of spread in ovarian cancer, occurring in approximately 14–70 % of patients, mainly in the pelvic and para-aortic region [4]. Since 2014, the FIGO staging system has reclassified ovarian cancer with SILN metastasis as stage IVB [2], while patients exhibited metastatic retroperitoneal lymph nodes are classified as stage IIIC, even if the primary tumor is confined to the pelvic cavity. This reclassification has sparked ongoing debate regarding its prognostic value. SILN metastasis in ovarian cancer is rare [5,6], and often accompanied by extensive intra-abdominal dissemination; cases without such spread are even more uncommon. Autopsy studies indicate that inguinal lymph node metastasis occurs in 0–3 % of patients with advanced ovarian cancers [[4], [5], [6]], explaining why SILN dissection is not routinely performed as part of ovarian cancer staging or debulking surgery. These autopsy studies, however, often lack detailed clinical information and pathological features, as patients with SILN metastasis frequently have significant tumor burden and extensive intra- abdominopelvic dissemination at the time of death. Actually, the number of reported cases of ovarian cancer metastasizing to SILN is far away from this autopsy estimated incidence.

A comprehensive literature review revealed only 5 retrospective cohort studies addressing SILN metastasis in primary ovarian cancer [[7], [8], [9], [10], [11]], with a total of 51 published cases (Table 1). Of these, 40 cases initially presented with inguinal masses. However, most early reports lacked systematic retroperitoneal lymph node dissection, limiting the available clinicopathological information. Table 1 shows that the age of patients at presentation ranged from 34 years to 84 years (mean 57.46 years). Notably, 41 of 51 patients underwent exploratory or staging laparotomy, and most case reports involved extensive intra-abdominal dissemination. 19 cases had no abnormalities in the abdominopelvic cavity apart from ovarian masses, indicating the occurrence of isolated SILN metastasis. Normal-sized ovarian cancer syndrome with SILN metastasis is exceedingly rare. Manci et al. [12] reported a 58-year-old patient presenting solely with bilateral SILN enlargement. The results of preoperative gastroscopy, ultrasonography, and abdominal CT were negative, but PET-CT showed an increased uptake of 18F-FDG in the inguinal and both adnexal areas. Then exploratory laparotomy revealed no masses in the abdominopelvic cavity and both adnexa, but postoperative pathological diagnosis confirmed low-grade differentiation serous papillary adenocarcinoma of bilateral ovarian and metastatic bilateral inguinal lymph nodes, without any intraperitoneal or lymphatic spread. Scholz et al. [13] reported a case of undifferentiated serous adenocarcinoma of bilateral ovaries, initially displayed an isolated left SILN metastasis, with involvement of the fimbria of right fallopian tube. Pathology of peritoneal washing showed papillary adenocarcinoma cells, but the pelvis and abdomen were otherwise normal, with negative para-aortic and pelvic lymph nodes. These rare cases demonstrate that ovarian tumor cells can metastasize directly to the inguinal region, even in relatively early stages of tumor growth. Kehoe et al. [14] reported a case where left SILN metastasis of poorly differentiated adenocarcinoma preceded the diagnosis of left ovarian poorly differentiated adenocarcinoma with extensive intra-abdominal dissemination by 33 months. The patient in our case report also had no detectable ovarian tumor at initial diagnosis, highlighting the importance of close follow-up after bilateral adnexectomy and adjuvant chemotherapy.

**Table 1:** Clinicopathologic Feature of all reported cases of primary ovarian cancer with inguinal node metastasis.

First Author	Year of publication	Number of cases	Age	CA125 U/ml	Side	Tumor histology	Peritoneal Disease status	Other nodes involved	Treatment	Follow up (month)
Ling-li Zhang [21]	2023	1	47	1381.6	Ipsilateral	HGSC	Minimal	right iliac LN positive	Surgery/chemo	NED
Ying Zhao [22]	2023	1	68	922.4	Ipsilateral	HGSC	Bulky	negative	Surgery/chemo	DFS, 20
Eliška [23]	2022	1	58	//	Ipsilateral	HG endometroid carcinoma	Minimal	negative	Surgery/chemo	DFS, 12
Dam [24]	2021	1	62	3628	//	HGSC	Minimal	unresected	Chemo/avastin	Relapse of coeliac trunk and hepatic artery LN, 2 years
Bacalbasa [25]	2018	1	46	67	Ipsilateral	adenocarcinoma	Minimal	1 pelvic LN positive	Surgery/chemo	DFS, 12
Moro [26]	2018	1	50	>5000	Ipsilateral	HGSC	Minimal	pelvic,para-aortic,cardio-phrenic angle LN positive	Surgery	//
Metwally [27]	2017	4	53	533	Ipsilateral	HGSC	Bulky	negative	Surgery/chemo	Relapse 12, AWD 48
			59	//	Ipsilateral	HGSC	Bulky	negative	NACT/surgery/chemo	during chemo
			65	233	Ipsilateral	HGSC	Bulky	negative	NACT/surgery/chemo	during chemo
			62	341	Ipsilateral	HGSC	Bulky	negative	Surgery/chemo	NED
Weizhang Shen [28]	2017	1	51	>500	Ipsilateral	serous adenocarcinoma	Bulky	unresected	Chemo	AWD, 24
Haidopoulos [19]	2016	1	34	//	Contralateral	//	//	//	//	//
Yang [29]	2014	1	54	正常	Ipsilateral	HGSC	Minimal	negative LN, positive cytology of the pelvic fluid	Surgery/chemo	DFS, 60
Deka [30]	2013	1	35	412	Ipsilateral	LGSC	Minimal	unresected	NACT/surgery/chemo	DFS, 24
Lee [15]	2010	1	46	260.01	Ipsilateral	malignant melanoma	Bulky	retroperitoneal,para-aortic LN positive	Surgery/chemo	DOD, 2
Oei [31]	2008	1	49	477	Ipsilateral	HGSC	Bulky	unresected	Surgery/chemo	NED
Xiaojun Yang [32]	2008	1	54	//	Ipsilateral	HGSC	Minimal	negative LN, positive cytology of the pelvic fluid	Surgery/chemo	DFS, 36
Rui Lu [33]	2008	1	67	//	Ipsilateral	HGSC	Bulky	//	//	//
Ang [18]	2007	1	59	215	Contralateral	moderately differentiated SC	Minimal	unresected	Surgery/chemo	DFS, 13
Manci [12]	2006	1	58	normal	Ipsilateral	HGSC	Minimal	negative	Surgery/chemo	DFS, 24
Diaz-Montes [34]	2005	1	49	3150	Ipsilateral	HGSC	Bulky	14 pelvic,4 para-aortic LN positive	Surgery/chemo	DFS, 32
Euscher [8]	2004	20	49	//	//	HGSC	Bulky	negative	Surgery/chemo	NED, 12
			61	//	//	HGSC	Bulky		Surgery/chemo	DOD, 36
			67	//	//	HGSC	Minimal		Surgery/chemo/ radiation	AWD, 46
			83	//	//	HGSC	//		//	//
			61	//	//	LGSC	Minimal		Surgery/chemo	NED, 25
			52	//	//	HGSC	Bulky		Surgery/chemo	DOD, 9
			60	//	//	HGSC	Minimal		Surgery/chemo/ radiation	NED, 12
			68	//	//	HGSC	Minimal		Surgery/chemo	DOD, 26
			61	//	//	HGSC	Bulky		Surgery/chemo	DOD, 108
			50	//	//	HGSC	Minimal		Surgery/chemo	AWD, 46
			61	//	//	HGSC	Bulky		Surgery/chemo	AWD, 78
			70	//	//	Not graded	Bulky		Chemo	DOD, 22
			62	//	//	HGSC	Bulky		Chemo	DOD, 24
			63	//	//	HGSC	Bulky		Chemo	DOD, 4
			46	//	//	HGSC	Bulky		Surgery/chemo	NED, 6
			85	//	//	LGSC	Bulky		Surgery/chemo	NED, 74
			50	//	//	HGSC	Minimal		Surgery/chemo	NED, 40
			47	//	//	HGSC+LGSC	Bulky		Surgery/chemo	NED, 5
			75	//	//	HGSC	Minimal		Surgery/chemo	DOD, 27
			84	//	//	HGSC	Minimal		//	AWD, 6
Scholz [13]	1999	1	43	//	Ipsilateral	HGSC	Minimal	negative LN, positive cytology of the pelvic fluid	Surgery/chemo	DFS, 3
Dihong Tang [35]	1999	4	46	1049	Ipsilateral	HGSC	Bulky	para-aortic LN positive	Surgery/chemo	DFS, 36
			65	328.8	Ipsilateral	HGSC	Bulky	Unresected, with left supraclavicular LN metastasis	NACT/surgery/chemo	DFS, 36
			48	324.1	Ipsilateral	HGSC	Bulky	unresected, with left supraclavicular LN metastasis	NACT/surgery/chemo	Relapse of left supraclavicular LN, 17 months
			54	//	Ipsilateral	HGSC	Bulky	unresected	Surgical exploration	DOD, 2
Triolo [36]	1996	1	//	//	//	SC	//	//	//	//
Chen [17]	1995	1	48	1024	Contralateral	moderately differentiated SC	Minimal	15 pelvic,2 para-aortic,5 scalene LN positive	Surgery/chemo	DOD, 14
Kehoe [14]	1993	1	66	480	Ipsilateral	HGSC	Bulky	unresected	Surgery/chemo	DFS, 24
McGonigle [37]	1992	1	59	205	Ipsilateral	EC	Minimal	unresected	Surgery/chemo	DFS, 32
Shulman [16]	1953	1	63	//	Contralateral	SC	Minimal	unresected	Surgery/radiation	DFS, 6 weeks

side (in relation to the ovarian cancer); HG, high-grade; LG, low-grade; SC, serous adenocarcinoma; EC, endometroid carcinoma; LN, lymph node; Chemo, chemotherapy; NACT, neoadjuvant chemotherapy; NED, no evidence of disease; DOD, dead of disease; AWD, alive with disease; DFS, disease free survival; //, Information not provided.

Aside from one case of ovarian malignant melanoma reported by Lee et al. [15], the primary ovarian cancer of all published cases were epithelial types, mostly high-grade (poorly differentiated) serous adenocarcinomas. Except for cases reported by Euscher et al [8] without detailed clinical information, most reported cases suffered from ipsilateral inguinal metastasis in relation to the ovarian cancer, while contralateral SILN metastasis has been documented in only 4 cases [[16], [17], [18], [19]]. Shulman et al. [16] reported the first case of primary ovarian cancer with contralateral SILN metastasis in 1953. Researchers have speculated that lesions may metastasize to the contralateral SILN via lymphatic channels near the uterine fundus or cervix, or through hematogenous spread [20].

### Pathways and mechanisms of SILN metastasis in ovarian cancer

Scholz et al. [13] proposed that the primary lymphatic drainage pathway of ovarian cancer follows the infundibulopelvic ligament toward the para-aortic and para-cava regions. When this pathway is obstructed (e.g., by tumor thrombi), tumor cells may undergo retrograde lymphatic drainage to the pelvic region and SILNs. Bacalbasa et al. [38] reported a case of ovarian cancer presenting with bilateral inguinal masses, diagnosed by PET-CT, which showed pathologically negative para-aortic lymph nodes but positive pelvic lymph node (1) and SILN (18) involvement, supporting this hypothesis.

Kleppe et al. [20] investigated the lymphatic drainage pathways of the human ovaries and anatomical drainage regions of sentinel lymph nodes using immunohistochemical analysis and three-dimensional reconstruction of pelvic lymphatics in 3 human female fetuses with an embryonic age of 14, 15, and 20 weeks, thus verify the rarity of inguinal metastases. Two major and 1 minor lymphatic drainage pathways from the ovaries were detected. Firstly abdominal pathway: Lymph vessels drain the ovaries at its cranial part following the course of ovarian artery within the infundibulopelvic ligament, then entered the retroperitoneal space and traveled along the common iliac artery and aorta on the left side, and the common iliac artery and caval vein on the right side. Secondly pelvic pathway: Lymphatic drainage ran from the caudal part of the ovaries downwards to the proper ligament of the ovary (ovarian ligament), followed the ovarian-uterine branch of the uterine artery along the whole corpus uterus towards to the level of the cervix of the uterus, then run along the course of the uterine artery, crossing the ureter superiorly, draining into the lymph plexuses surrounding the internal iliac artery and obturator fossa. Thirdly minor inguinal pathway: A few lymphatic vessels within the round ligament of the uterus to the distal external iliac and SILNs. The study showed that the inguinal pathway was more prominent in the fetuses of 14 and 15 weeks compared to the fetus of 20 weeks. In the fetus of 15 weeks, lymphatic vessels were observed in both round ligaments, with the lymphatics on the left side abruptly disappearing when traveling more caudally and the lymphatics on the right side becoming thinner toward the inguinal region and eventually drained into SILN. Meanwhile the study confirmed the presence of lymph vessels in the infundibulopelvic ligament, proper ligament of the ovary (ovarian ligament), and round ligament of the uterus of the human adult cadaveric tissues samples. The authors speculated that the inguinal pathway for ovarian cancer lymphatic metastasis may disappears during embryogenesis in the majority of female fetuses, but may persist into adulthood in a small percentage, forming a potential route for lymphatic spread of ovarian cancer.

Togami et al. [39] reported a 44-year-old ovarian cancer patient who underwent optimal debulking surgery and adjuvant chemotherapy, presenting with a left inguinal mass as a recurrence two years later. Pathology of the excised mass showed serous adenocarcinoma originating from the left round ligament without involvement of the left SILN, further supporting the inguinal pathway for ovarian cancer lymphatic metastasis. Whether surgical extirpation of the primary drainage areas (para-aortic and iliac) would re-open obliterated lymphatic to inguinal nodes is unknown. Metwally et al. [27] reported a patient of ovarian cancer with metastatic inguinal lymphadenopathy, who complained a history of right ureteric resection and reconstruction by segment of ileum, speculating that previous surgery may have disrupted the lymphatic drainage of iliac and para-aortic regions, leading to the reconstruction of the lymphatic drainage in inguinal region.

Hematogenous metastasis occurs in approximately 2–3 % of patients with primary ovarian cancer [40] and theoretically may account for dissemination to any distant sites in ovary cancer, such as central nervous system, breast, cardiophrenic angle lymph nodes, and axillary lymph nodes [20,26,41], as well as contralateral SILN metastasis. Therefore, we propose that isolated SILN metastasis in ovarian cancer potentially involve two pathways: via deep inguinal lymphatic routes through round ligament and/or hematogenous route. This unique metastatic pattern likely reflects tumor biology and host immune status. The special host immune state within a specific time window perhaps plays a crucial role, might recognize and kill some primary cancer cells but neglect metastatic tumor foci such as distant isolated lymph node metastasis. Existing literatures have put more emphasis on pelvic and para-aortic lymph nodes metastasis in ovarian cancer. However, few studies had focused on the exact molecular mechanisms and/or risk factors of the isolated SILN metastasis in ovarian cancer, still deserving further investigation.

### Prognosis of ovarian cancer with SILN metastasis

Lymph node metastasis is generally recognized as a poor prognostic factor in ovarian cancer [42]. However, the impact of distant lymph node metastasis on survival of stage IV ovarian cancer prognosis remains controversial over the years. Some researchers suggested that patients with SILN metastasis were associated with poor prognosis [9,17], while others argued that complete optimal secondary debulking surgery is feasible for recurrent ovarian cancer with isolated lymph node metastasis (including SILN involvement), associated with relatively favorable survival rates [43,44]. Reports indicate disease-free survival of up to 5 years in primary ovarian cancer patients with isolated SILN metastasis [29].

According to the updated FIGO staging system, ovarian cancer with SILN metastasis was reclassified into stage IVB from stage IIIC, rasing questions about its staging value and the prognostic impact of distant lymph node metastasis. Euscher et al. [8] conducted a retrospective study showing that distant lymph node metastasis (including 20 cases of SILN metastasis) in serous ovarian cancer, although uncommon, did not adversely affect survival. Patients with adenopathy and minimal peritoneal disease (grossly negative omentum) had a median survival of 120 months compared with 24 months for those with bulky peritoneal disease (grossly positive omentum). Thus, patients with minimal peritoneal metastasis and extra-abdominal lymph node metastasis had longer survival than those with bulky peritoneal metastasis. Hjerpe et al. [45] studied 551 patients with stage IV serous ovarian cancer in Sweden between 2009-2014 in order to explore the prognostic impact of isolated distant lymph node metastasis as only stage IV classifier in serous ovarian cancer. The results showed that median overall survival for patients with lymph node-only positive (n=51) was 41.4 months, compared to 25.2, and 26.8 months for patients with pleural involvement (n=195), or other/multiple (n=187) distant metastases (p=0.0007). The corresponding five-year survival rates were 32 %, 11 %, and 22 %, respectively. Multivariate analysis confirmed shorter survival for patients with pleural involvement (HR=2.99, p=0.001) or other/multiple distant sites (HR=2.67, p=0.007) as compared to the lymph node-only positive group. In consequence patients with stage IV serous ovarian cancer having lymph nodes as only distant metastatic site live longer than other stage IV patients.

Nasioudis et al. [11] retrospectively analyzed the five-year overall survival (OS) of 11,152 patients diagnosed with epithelial ovarian cancer from the Surveillance, Epidemiology, and End Results database (2004-2013). The results showed five-year OS for patients in group 1 (n = 151, stage IV due to positive SILN) was 46.3 %, compared to 44.9 % for those in group 2 (n = 4403, stage III with positive para-aortic/pelvic nodes) (p =0.4), 32.9 % in group 3 (n = 642, stage IV with positive distant lymph nodes only) (p <0.001) and 25.3 % in group 4 (n = 5956, stage IV with other distant metastases) (p <0.001). In conclusion, patients with stage IV ovarian cancer due solely to inguinal nodal metastases have similar survival as those with pelvic/para-aortic nodal involvement and improved survival compared to those harboring distant metastases. The higher survival rates in patients with isolated SILN metastasis may be attributed to their specific immune environment.

### Diagnosis and treatment of ovarian cancer with SILN metastasis

Due to its insidious onset, ovarian cancer usually presents with advanced stage at their first diagnosis, with symptoms and signs related to extensive intraperitoneal metastases, such as abdominal pain, distension, and macritus. When encountering patients with asymptomatic isolated inguinal masses at the time of first visit, comprehensive diagnostic assessments are essential. Zaren et al. [46] retrospectively studied 2,232 patients with pathologically confirmed SILN metastatic cancer, noting that these tumors could originate from various sites, including breast cancer, the vulvar and lower third of the vagina, pelvic malignancies, anal squamous cell carcinoma, rectal cancer, skin malignancies such as malignant melanoma, or squamous cell carcinoma of the legs and trunk. The most common primary tumor was melanoma (27 %), while ovarian cancer accounted for only 5 % of cases. Systematic infectious disease such as syphilis, HIV, herpes simplex virus infection, and local infections like ulcers in the lower genital tract or gonorrhea can also cause SILN enlargement. For patients with painless lymph node enlargement, malignant diseases should be considered. Fine-needle aspiration or biopsy of the lymph node can be the important investigation to determine its nature, and immunohistochemistry can help to identify the primary tumor origin.

For patients with isolated SILN metastasis of unknown origin, laparoscopic exploration, as a minimally invasive procedure, has significant value in the diagnosis of pelvic and abdominal tumors, although small tumors within the ovary might be missed [47]. Imaging examination such as transvaginal ultrasound, CT, and MRI are critical in detecting the earlier malignancy in ovary. PET-CT is particularly helpful in the diagnosis of occult ovarian cancer, even when CA125 level is within the normal range [12,25]. Manci [12] and Bacalbasa [12] each reported a case of ovarian cancer presenting with inguinal metastatic masses that could only be diagnosed through PET-CT. Therefore, a detailed case history collection, complete gynecological examination and some useful auxiliary diagnostic measures for any ovarian neoplasms are necessary for the management of a patient presenting with inguinal enlargement of unknown origin. Even in the absence of evident clinical symptoms, sign and supportive auxiliary examination results for ovarian cancer, isolated enlarged SILN should also be paid enough attention for possible existence of an occult ovarian cancer [14].Patients with cancer of unknown origin (CUP) are categorized into two prognostic subgroups, according to their clinicopathologic characteristics. The favorable risk cancer subgroup (15–20 %) includes patients with peritoneal adenocarcinomatosis of a serous papillary subtype, and isolated axillary nodal metastases in females [48]. In the era of targeted therapies, accurate histopathological and molecular classification of tumors is essential for CUP. Classifications based on epigenetic alterations have served this purpose. The DNA-methylation-based classifier EPICUP has enabled the correct identification of the tumour of origin in a higher proportion of patients with CUP compared with previously established approaches (such as immunohistochemical analysis and image-assisted technologies), with more confidence than other gene-expression-based platform [49].

SILN metastasis in ovarian cancer patients often indicates advanced disease and dissemination, with a poor prognosis. The standard treatment for ovarian cancer confined to the pelvis and abdomen remains optimal cytoreductive surgery combined with platinum-taxane chemotherapy, with/ without maintenance therapy of bevacizumab and/ or poly (ADP-ribose) polymerase (PARP) inhibitors. Currently, new biological agents are emerging from recent clinical trials, among which several drugs have been incorporated into the standard treatment. The activation of PI3K/Akt/mTOR pathway, which is the most often aberrant signalling pathway discovered in 70 % of ovarian cancer cases, is linked to aggressive phenotypes, chemoresistance, and a poor prognosis, making it an important therapeutic target named PI3K/AKT/mTOR inhibitor [50]. For cases with isolated SILN metastasis, combining surgical treatment with chemotherapy may yield a relatively favorable prognosis [10,29]. For these patients, whether primary or recurrent, optimal cytoreductive surgery seems to achieve good therapeutic effects and survival benefits [29,43]. There is still controversy regarding the timing of surgery and whether neoadjuvant chemotherapy is necessary. Giri et al. [10] conducted a retrospective study of 7 ovarian cancer patients with SILN metastasis, showing that neoadjuvant chemotherapy had a good response in patients with extensive pelvic and abdominal metastases, with one case even achieving complete remission during interval debulking surgery. While the effectiveness of chemotherapy and surgery for these patients is clear, the optimal sequence remains debatable. Hjerpe et al. [45] reported 551 stage IV serous ovarian cancer patients, among which 65 % underwent primary debulking surgery and 35 % underwent interval debulking surgery, with no significant difference in survival between the two groups. For stage IV patients with only distant lymph node positivity, those who underwent primary debulking surgery (n=22) had a 9.1-month longer overall survival compared to those who underwent interval debulking surgery (n=20), but the difference was not statistically significant (p=0.245), possibly due to the small sample size.

Clinically, we need to be vigilant about SILN metastasis in ovarian cancer. When performing systematic retroperitoneal lymphadenectomy, deep inguinal lymph nodes along the external iliac vessels should be routinely removed. In cases with positive deep inguinal lymph nodes, attention should be paid to SILN enlargement. Currently, there is a lack of standardized diagnosis and treatment guidelines for ovarian cancer patients with isolated SILN metastasis. Large-scale, multicenter studies are needed to investigate the biological and clinical behavior of tumors in patients with SILN metastasis and to explore the clinical outcomes of stage IVB ovarian cancer patients with disease limited to the ovary but with SILN metastasis.

## Conclusion

Isolated SILN metastasis can occur as an initial symptom in rare case of ovary cancer, even at relatively early tumor stages, mediated through the inguinal lymphatic and (or) hematogenous route under special immunological, pathological and hormonal conditions. This highlights the importance of considering inguinal masses as potential indicator of ovarian malignancy. When SILN enlargement is observed, especially with elevated CA125 levels, ovarian cancer should not be overlooked. Comprehensive preoperative assessments, including SILN biopsy, thorough gynecological examination, and necessary auxiliary investigations, are crucial for accurate diagnosis [29]. The potential mechanisms, risk factors, and metastatic patterns of isolated SILN metastasis in ovarian cancer remain unclear and require further investigation through large-scale, multicenter studies to elucidate these aspects and develop optimal management strategies for this unique presentation of ovarian cancer.

## Funding

This study was funded by the "Spearhead and Leading wild goose" Research Project(2024C03145) of Zhejiang Province, China.

## CRediT authorship contribution statement

**XiaoJing Guan:** Writing – original draft, Project administration, Data curation. **Zhi Ma:** Writing – review & editing, Validation. **JianHua Yang:** Writing – review & editing, Supervision.

## Declaration of competing interest

The authors declare that they have no known competing financial interests or personal relationships that could have appeared to influence the work reported in this paper.
